# Supplementary figures and images for: Probing the effect of PEG-DNA interactions and buffer viscosity on tethered DNA in shear flow
Source: PLoS One. 2025 Aug 25;20(8):e0329961. doi: 10.1371/journal.pone.0329961 (PMC12377632; doi:10.1371/journal.pone.0329961)

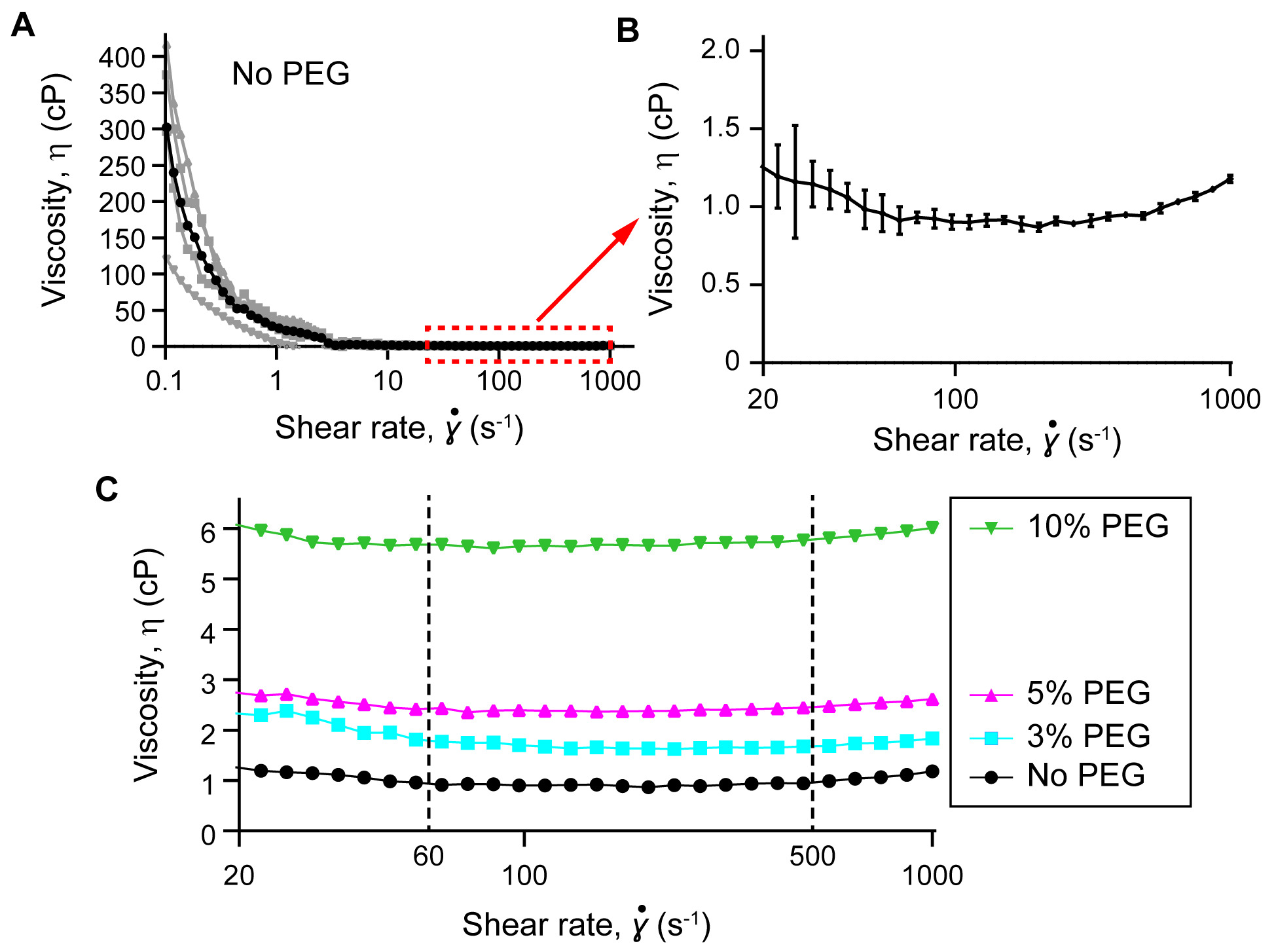

Supplement: S1 Fig — (A) Buffer viscosity η in the absence of PEG. Shown are the results for ηin units of centipoise (cP) for four measurements (gray) and the average of these measurements (black) (1cP=1mP·s). (B) For better visibility, shown is the average of ηin (A) for shear rates γ˙ in the range 20–1000 s−1 as indicated by the red box in (A). Error bars: SD. (C) Buffer viscosity η in the absence of PEG and with 3%, 5%, and 10% concentrations of PEG, respectively, for shear rates γ˙ in the range 20–1000 s−1 obtained as the average of five measurements for each concentration of PEG. Shear rates were averaged over the range 60–500 s−1 for subsequent analysis in this study. (TIF) [file pone.0329961.s001.tif]
